# Tissue content of metalloproteinase-9 and collagen in the colon with and without fecal stream after intervention with infliximab in rats subjected to Hartmann’s surgery

**DOI:** 10.1590/ACB360401

**Published:** 2021-05-14

**Authors:** Antonio José Tiburcio Alves, José Aires Pereira, Mariane Grandi de Ávila, Fernanda Aparecida Domingues, Daniela Tiemi Sato, Carlos Augusto Real Martinez

**Affiliations:** 1 Fellow PhD degree. Universidade Estadual de Campinas – Faculty of Medical Sciences – Postgraduate Program in Surgical Sciences – Campinas (SP), Brazil.; 2 PhD, Assistant Professor. Universidade São Francisco – Faculty of Medicine – Division of Pathology – Bragança Paulista (SP), Brazil.; 3 Graduate student. Universidade São Francisco – Faculty of Medicine – Department of Pathology – Bragança Paulista (SP), Brazil.; 4 Fellow Master degree. Universidade Estadual de Campinas – Faculty of Medical Sciences – Postgraduate Program in Surgical Sciences – Campinas (SP), Brazil.; 5 PhD, Associate Professor. Universidade São Francisco – Faculty of Medicine – Postgraduate Program in Health Sciences – Bragança Paulista (SP), Brazil.

**Keywords:** Colitis, Volatile Fatty Acids, Tumor Necrosis Factor-alpha, Collagen, Matrix Metalloproteinase 9, Rats, Free Oxygen Radicals

## Abstract

**Purpose:**

Quantify the tissue content of metalloproteinase-9 (MMP-9) and collagen in colic mucosa with and without intestinal transit after infliximab administration in rats subjected to Hartmann’s surgery.

**Methods:**

Twenty-two rats underwent colon diversion by Hartmann’s surgery. Animals were maintained with intestinal bypass for 12 weeks to induce development of diversion colitis (DC). Afterwards, animals were divided into three groups: first group received subcutaneous application of saline solution (SS) 0.9%, while the remaining two groups received infliximab subcutaneously at doses of 5 or 10 mg·kg^–1^·week^–1^ for five consecutive weeks. After the intervention, animals were sacrificed, removing the segments with and without intestinal transit. Diversion colitis was diagnosed by histological study, and its intensity was determined by a validated inflammatory scale. Tissue expression of MMP-9 was assessed byimmunohistochemistry, while total collagen was assessed by histochemistry. Tissue content of both was measuredby computerized morphometry.

**Results:**

Colon segments without intestinal transit had a higher degree of inflammation, which improved in animals treated with infliximab. Collagen content was always lower in those without intestinal transit. There was an increase in the collagen content in the colon without transit in animals treated with infliximab, primarily at a dose of 10 mg·kg^–1^·week^–1^. There was an increase in the content of MMP-9 in the colon without fecal transit, and a reduction was observed in animals treated with infliximab, regardless of the dose used.

**Conclusions:**

Application of infliximab reduces inflammation, increases the total collagen content and decreases the content of MMP-9 in the colon without intestinal transit.

## Introduction

Ileostomy or colostomy, whether temporary or permanent, is a surgical procedure increasingly used for the management of several colorectal diseases, primarily represented by congenital anorectal anomalies, intestinal obstruction, intestinal inflammatory diseases (IBDs), complications of acute sigmoid diverticulitis, colorectal trauma, colorectal tumors and severe anoperineal infections^1^. However, in addition to the difficult physical and psychological adaptation to life with a stoma, a series of complications, either early or late, related to the procedure may arise that further reduce the quality of life of these patients. Among the late complications related to the construction of a stoma after Hartmann’s surgery (HS), the chronic inflammatory process that affects the exclusive segments of fecal transit stands out. Depending on the affected large intestine segment, this colorectal inflammatory process is known as proctitis or diversion colitis (DC)^2^^,^ 3.

Several hypotheses have been proposed to explain the etiopathogenesis of DC. However, currently, the most accepted theory relates the appearance of DC to short-chain fatty acids deficiency (SCFAs) due to the exclusion of intestinal transit^4^. A study using an experimental DC model showed that the deprivation of the main substrate for the normal metabolism of colic mucosa cells modifies the mitochondrial respiratory mechanisms, causing an increase in the production of free oxygen radicals (FOR)^5^. Free oxygen radicals are toxic substances to epithelial cells due to their high oxidative and proinflammatory power and they destroy the main defense mechanisms that form the epithelial barrier of the colic mucosa^5^. Thus, DC has been considered a disease caused by energy deficiency resulting from the lack of a regular supply of SCFAs to colic epithelial cells due to diversion of the fecal stream^6^.

The importance of adequate DC treatment is becoming increasingly apparent when considering the large number of patients who live with a colon or rectum segment without intestinal transit. To date, the main therapeutic strategy for the treatment of DC has been to try to restore the supply of SCFAs to the colon epithelial cells by reconstructing intestinal transit^3^. However, this is not always possible, particularly after HS^7^. The reestablishment of intestinal transit after HS has high rates of morbidity and mortality, particularly related to anastomosis dehiscence performed in a dysfunctional and inflamed colic segment. In these patients, clinical treatment becomes the only feasible therapeutic strategy^8^^,^ 9.

Few studies have evaluated the collagen content in the excluded colon, as well as the behavior of other proteins involved in the healing process^10^^,^ 11. Collagen is considered one of the main components of the extracellular matrix (ECM), under which the other components are allocated^12^. On the other hand, metalloproteinases are collagenases with the potential for oxidation and reduction that promote the degradation of amorphous and fibrillar collagens and other components of the ECM^13^. Metalloproteinase-9 (MMP-9) is a protein that belongs to the group of gelatinases, whose main function is to degrade denatured collagen during inflammatory tissue processes^13^. Metalloproteinase-9 has been linked to the development of IBD; however, the importance of increasing the content of MMP-9 in colorectal inflammation is still poorly understood^14^. When considering the segments lacking intestinal transit that develop DC, it is possible that there may be less tissue production of collagen due to the lack of an energy supply and greater degradation due to the increased activity of MMP-9, formed by the largest inflammatory infiltrate^13^^,^ 14.

Tumor necrosis factor alpha (TNF-alpha) is a cytokine that acts directly in the acute phase of inflammatory processes, and its role is related to the balance of immune cell actions^15^^-^ 17. Infliximab, in turn, is a monoclonal antitumor necrosis alpha antibody (anti-TNF alpha) that acts by blocking the proinflammatory activity of TNF-alpha^13^. Recently, infliximab has been shown to be effective for the treatment of DC^15^. The antibody, in addition to favoring the epithelialization of the inflamed mucosa, reduced the inflammatory process and decreased the neutrophilic infiltrate in the mucosa excluded from intestinal transit^15^. It is possible that infliximab, by decreasing the mucosal inflammatory process and the infiltration of neutrophils in the excluded colon, may reduce the local production of MMP-9 and consequently decrease the degradation of tissue collagen.

However, the effects of infliximab on the tissue content of collagen and MMP-9 in segments without fecal transit that develop DC have not been studied to date. Thus, the aim of this study was to evaluate the effects of infliximab therapy on the total content of tissue collagen and MMP-9 in an experimental model of DC. It is also intended to evaluate the relationship between the infliximab dose used and the content of both proteins.

## Methods

This study was carried out in compliance with Federal Law 6,638 and the guidelines of the Brazilian College of Animal Experimentation (COBEA). The study was submitted to and approved by the Ethics Committee on the Use of Animals in Research at Universidade São Francisco (Process No. 0102262014).

### Surgical technique and experimental groups

Twenty-two male Wistar rats were used. On the day of the intervention, the animals were anesthetized with ketamine hydrochloride at a dose of 5 mg·kg^–1^ associated with xylazine hydrochloride at a dose of 60 mg·kg^–1^ administered intraperitoneally. The abdominal cavity was accessed through a median, longitudinal, infraumbilical incision 4 cm in length. The abdominal wall was opened byplanes, identifying the rectosigmoid transition defined by the Peyer plate. The left colon was sectioned 8 cm above the cranial end of the plate. In all animals, after sectioning the colon, the distal segment of the colon and rectum was catheterized and irrigated with 40 mL of 0.9% saline at room temperature to remove fecal residues present in the distal segment. Irrigation was completed when there was no more fecal waste leaving the animal’s anus. After irrigation, the caudal segment of the intestine was buried and fixed to the parietal peritoneum. The sectioned cranial colon (with fecal transit) was exteriorized as a colostomy in the left hypochondrium. After fixation of the cranial colostomy, the abdominal wall was closed with two suture planes.

After performing HS, the animals were isolated in individual cages for 12 weeks for the induction of DC. After this period, they were divided into three experimental groups according to the intervention to be carried out: group A - 0.9% saline solution (control), group B - infliximab at a dose of 5 mg·kg^–1^·week^–1^ and group C - infliximab at a dose of 10 mg·kg^–1^·week^–1^. In all animals, the intervention solutions were administered weekly by subcutaneous application to the cervical skin fold. Subcutaneous application was chosen considering the difficult intravenous access and the favorable response to subcutaneous infliximab in rats with experimental colitis showed by Triantafillidis *et al.* 18. The intervention solutions were administered for five consecutive weeks.

After five weeks of intervention with infliximab, all rodents were again anesthetized, using the same methodology previously described, for the removal of the colic segments with and without intestinal transit. After the extraction of the colon specimens, the animals were euthanized by intracardiac injection of a single lethal dose of thiopental (120 mg·kg^–1^).

### Histological analyses

The excised specimens were fixed in 10% formaldehyde for 72 h and were subsequently dehydrated in successively increasing concentrations of alcohol. After the process, clarification of the specimens in xylene was carried out. Then, the material was included in paraffin blocks and subjected to longitudinal cuts that were 4 μm thick to mount on slides. After assembly, the slides were stained using hematoxylin-eosin techniques (for analysis of the specimen histological changes), histochemistry (Masson trichrome coloration) and immunohistochemistry to identify tissue collagen and MMP-9 expression.

Each slide was read under a common optical microscope at a final magnification of 200×. The histological parameters were analyzed by a pathologist experienced in diseases of the digestive tract that was unaware of the colic segment analyzed, as well as the experimental group to which the animal belonged. To produce the inflammatory score, the following histological parameters were used on slides stained by the hematoxylin-eosin technique: epithelial loss, atrophy of the colic glands, vascular congestion and inflammatory infiltrate. A score ranging from 1 to 3 crosses was assigned to each parameter, according to the degree of alteration found (0 = none; + = mild; ++ = moderate and +++ = severe). Colons with and without fecal transit in each experimental group were analyzed separately. The value adopted for the colic segment analyzed (with or without intestinal transit) for each animal in the group was the average found after reading three histological fields where there were at least three intact and contiguous glands. The final value assigned to each colic segment analyzed (with and without fecal transit) in each of the experimental group SS 0.9%, infliximab 5 mg·kg^–1^·week^–1^ and infliximab 10 mg·kg^–1^·week^–1^) was the average value obtained by adding the values of each parameter.

### Masson’s trichrome technique

Masson’s trichrome technique was first proposed by the Discipline of Pathology at Universidade Estadual de Campinas. Initially, the slides were dewaxed and hydrated in xylene and decreasing concentrations of alcohol. Afterwards, they were washed for 5 min in running water, covered with Bouin’s solution and kept at room temperature for 24 h. After this period, they were washed in running water until the complete removal of Bouin’s dye. Then, they were washed in distilled water and stained with Weigart’s iron hematoxylin for 10 min. After this stage, the slides were washed again in running water for 10 min and then in distilled water. Then, they were stained with Biebrich scarlet solution for 5 min and washed again with distilled water. After this phase, they were differentiated by a solution of phosphotungstic-phosphomolybdic acid for 15 min and washed again in distilled water. Afterwards, they were stained with aniline blue solution for 10 min, washed in distilled water and passed through a 1% glacial acetic acid solution for 5 min. Then, they were washed again in distilled water, dehydrated, cleared and mounted on sheets and coverslips with resin.

### Immunohistochemical technique

To research the tissue expression of the MMP-9 enzyme, a methodology standardized by the Medical Research Laboratory of the University of São Francisco previously described was used^11^. Primary monoclonal anti-MMP-9 antibody (monoclonal mouse for MMP-9; Abcam, Cambridge, MA, USA) was used. Briefly, the histological sections obtained from the segments with and without fecal transit of the animals from the three experimental groups were placed on slides previously marked and identified by the experimental group, the colon site where the fragment was removed and the animal number. Afterwards, they were immersed in a 1:100 solution of Trilogy (Trilogy, Brand Cell Marque, Cod-920P-04, batch 1129101B) at a temperature of 95 °C in a water bath for 45 min. Then, they were transferred to a second vat containing Trilogy solution at the same temperature, where they remained for 10 min. After this stage, the slides were removed and kept at room temperature for 30 min, after which they were washed in distilled water and phosphate buffered saline (PBS) for two minutes. The blocking of endogenous peroxidases was carried out with 10 V H_2_O_2_ for 10 min at room temperature. Afterwards, the slides were washed again in distilled water and PBS. To block nonspecific protein binding, the slides were exposed for 30 min to skimmed milk solution (Molico, Nestlé do Brazil, São Paulo, SP) and then washed again in distilled water and PBS. The primary anti-MMP-9 monoclonal antibody was used at a 1:100 dilution, and 100 μL of the diluted primary antibody was added to the sections and they were kept in a humid chamber for 1 h at room temperature. Then, they were washed with distilled water and twice with PBS for 2 min each. At the end of this step, incubation with the avidin-biotin system of the LSAB + System-HRP Kit (Dako do Brazil, São Paulo, SP; Reference K0690, batch 10068233) was carried out for 35 min with each reagent. After this period, the slides were washed with PBS twice and developed with the DAB + Substrate Liquid Kit (Dako do Brazil, São Paulo, SP; Reference K3468, batch 10066912) in the dilution of a drop of chromogen in 1 mL of buffer solution, where 100 μL of the chromogen was added to the slides and they were incubated for 5 min at room temperature. After development, they were washed with running water and stained with Harris’ hematoxylin for 30 s. After this stage, the slides were washed again with running water until thetotal removal of the excess hematoxylin and, finally, dehydrated through graded alcohol and cleared in xylene to be assembled with coverslips and resin. To standardize the results, the entire immunohistochemical technique was performed on a single day.

As directed by the primary antibody manufacturer, the positive control was performed on a specimen of normal liver tissue, while the negative control was performed on the same tissue without the addition of the primary anti-MMP-9 antibody.

### Measurement of tissue collagen content and MMP-9

In addition to the analysis of the inflammatory score, the content of tissue collagen and the enzyme MMP-9 in segments with and without intestinal transit was always performed in a place where there were at least three contiguous and intact colic glands. The selected image was captured by a video camera previously attached to an optical microscope (Eclipse DS50 - Nikon Inc., Japan). The image was processed and analyzed by the NIS-Elements program (Nikon Inc., Japan) installed on a computer with good image processing capacity. The measurement of the tissue content of both proteins was always performed at a final magnification of 200×. The image analysis program, using color histograms, determined the color intensity of each area selected for measurement, transforming the chosen color into a percentage numerical expression for each selected field of view. The final value adopted for each field measured in the colon with and devoid of transit was represented by the average of the values found after the evaluation of three different fields. For the quantification of collagen through the color histogram in the RGB system (red, green, blue), the blue color was selected, whose intensity was captured by the number of pixels containing the color and later converted into a numerical value (pixels·field^–1^). The same methodology was used to measure the content of MMP-9; however, the dark brown color was identified by immunohistochemistry.

### Statistical analysis

Descriptive statistics were used to calculate the values found after the measurements of each variable in each colic segment (with and without fecal transit) and in each experimental group (SS 0.9%, infliximab 5 mg·kg^–1^·week^–1^ and infliximab 10 mg·kg^–1^·week^–1^), and the results were expressed as the mean value and respective standard error. To evaluate the pattern of sample distribution, the Kolmogorov–Smirnov test was used. To compare all variables (inflammatory score, total collagen content and MMP-9) in the different colic segments and among the experimental groups, the Mann–Whitney nonparametric test was used, adopting a significance level of 5% (p < 0.05). The significant results obtained when comparing, in a paired way, the values obtained in the colon with and without fecal transit from the same experimental group were marked with an asterisk (*) when the p-value was less than 5% (p < 0.05) and with two asterisks (**) when it was less than 1% (p < 0.01). The significant results obtained when comparing, in a paired way, the values obtained in the animals submitted to intervention with SS 0.9%, infliximab 5 mg·kg^–1^·week^–1^ and infliximab10 mg·kg^–1^·week^–1^, within the same colic segment (with or without intestinal transit) were marked with a cross (†) when the p-value was less than 5% (p < 0.05) and with two crosses (††) when less than 1% (p < 0.01).

## Results

**Figure 1:**
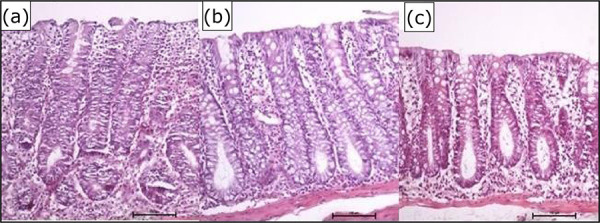
Colic mucosa with intestinal transit after intervention with **(a)** 0.9% SS, **(b)** infliximab 5 mg·kg^–1^·week^–1^,**(c)** infliximab 10 mg·kg^–1^·week^–1^.

Figure 1a-c shows the colic mucosa with intestinal transit after intervention with SS 0.9%, infliximab 5 mg·kg^–1^·week^–1^ and infliximab 10 mg·kg^–1^·week^–1^, respectively, for a period of 5 weeks. The colon of animals with maintained fecal transit and given an intervention with 0.9% SS or infliximab (in both doses), presented with preserved mucosa integrity, intestinal crypts with a normal distribution pattern, preservation of the population of goblet cells, structured histological layers and an absence of signs of inflammation, fibrosis and inflammatory cells.

**Figure 2:**
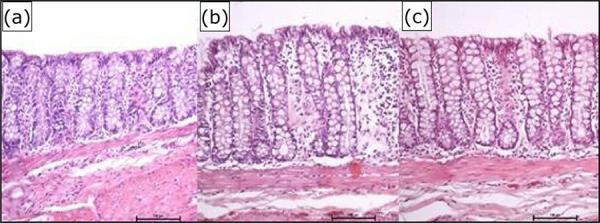
Colic mucosa without intestinal transit after intervention with **(a)** SS 0.9%, **(b)** infliximab 5 mg·kg^–1^·week^–1^,**(c)** infliximab 10 mg·kg^–1^·week^–1^.

Figure 2a-c shows the colic mucosa devoid of intestinal transit after intervention with SS 0.9%, infliximab5 mg·kg^–1^·week^–1^ and infliximab 10 mg·kg^–1^·week^–1^, respectively, for a period of five weeks. In the distal colon, with intestinal transit deprivation, from the animals of the control group submitted to intervention with SS 0.9%, a reduction in the height and architecture of the crypts was observed, there was a breakdown in the distribution and alignment of the colic glands, a decrease in the thickness of the mucous layer and loss of continuity between colonocytes. In contrast, in the distal colon excluded from animals that received an intervention with infliximab, the colic mucosa was more structured, with adequate thickness, intestinal crypts with a normal distribution pattern and a larger population of goblet cells, in addition to colonocytes arranged in parallel with continuity junctions.

**Figure 3:**
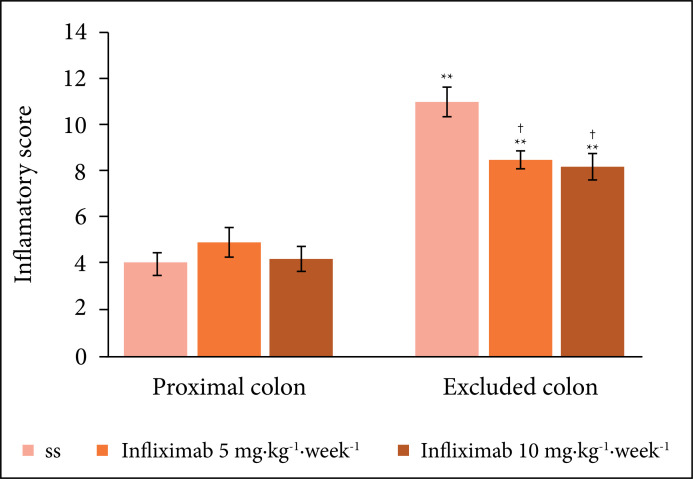
Inflammatory score in the proximal and excluded colon in animals submitted to intervention with 0.9% SS or infliximab at concentrations of 5 or10 mg·kg^–1^·week^–1^. ** = p < 0.01 (excluded colon > proximalcolon). † = p < 0.05 (infliximab 5 and 10 mg·kg^–1^·week^–1^< SF 0.9%). Mann–Whitney test.

Figure 3 compares the inflammatory score in the colon with and without intestinal transit, comparing animals submitted to intervention with SS 0.9% or infliximab at concentrations of 5 or 10 mg·kg^–1^·week^–1^.

**Figure 4:**
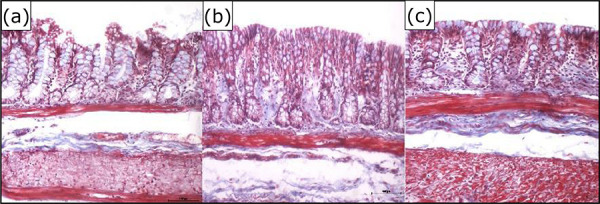
**(a)** Collagen in the colic mucosa devoid of intestinal transit after intervention with SS 0.9%. **(b)** Collagen in the colic mucosa devoid of intestinal transit after intervention with infliximab 5 mg·kg^–1^·week^–1^. **(c)** Collagen in the colic mucosa devoid of intestinal transit after intervention with infliximab 10 mg·kg^–1^·week^–1^ (Masson’s trichrome 200×).

Figure 4a-c show the tissue expression of total collagen in the colic mucosa devoid of fecal transit submitted to intervention with SS 0.9% or infliximab at doses of 5 and 10 mg·kg^–1^·week^–1^, respectively, during the same period.

**Figure 5:**
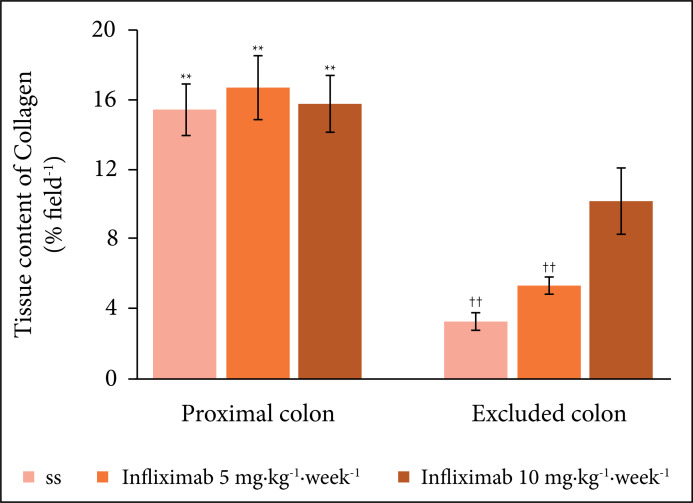
Tissue collagen content in animals given a weekly intervention with SS 0.9% and infliximab at dosages of 5 and 10 mg·kg^–1^·week^–1^ for five weeks. Note. ** p < 0.01 (proximal colon > excluded colon). †† p < 0.01 (SS < infliximab 5 mg·kg^–1^·week^–1^ and infliximab 5 mg·kg^–1^·week^–1^ < infliximab 10 mg·kg^–1^·week^–1^). Mann–Whitney test.

Figure 5 shows the values found for tissue collagen content in animals given weekly interventions with SS 0.9% and infliximab at dosages of 5 and 10 mg·kg^–1^·week^–1^ for five weeks. It appears that there is a significant increase in collagen content in animals treated with infliximab when compared to those receiving SS 0.9% and, in animals treated with infliximab at a concentration of 10 mg·kg^–1^·week^–1^, there was a greater increase when compared to those treated with infliximab at a concentration of5 mg·kg^–1^·week^–1^.

**Figure 6:**
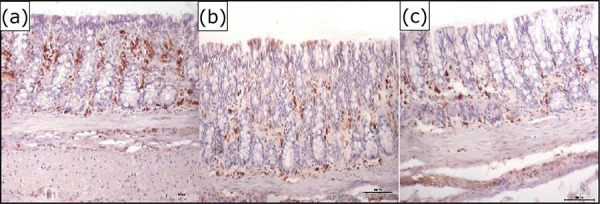
**(a)** Expression of the MMP-9 enzyme in the colic mucosa devoid of intestinal transit after intervention with 0.9% SS. **(b)** Expression of MMP-9 in the colic mucosa devoid of intestinal transit after intervention with infliximab 5 mg·kg^–1^·week^–1^. **(c)** Expression of MMP-9 in the colic mucosa devoid of intestinal transit after intervention with infliximab 10 mg·kg^–1^·week^–1^ (HI-anti-MMP9 200*×*).

Figure 6a shows the colic mucosa without fecal transit subjected to intervention with 0.9% SS for five weeks, while Fig. 6b and c show the mucosa treated with infliximab at doses of 5 and 10 mg·kg^–1^·week^–1^, respectively, for the same period. It was found that, in the excluded colon treated with SS 0.9%, there was a greater expression of MMP-9 in the colic mucosa compared to the animals submitted to the intervention with infliximab at both concentrations.

**Figure 7:**
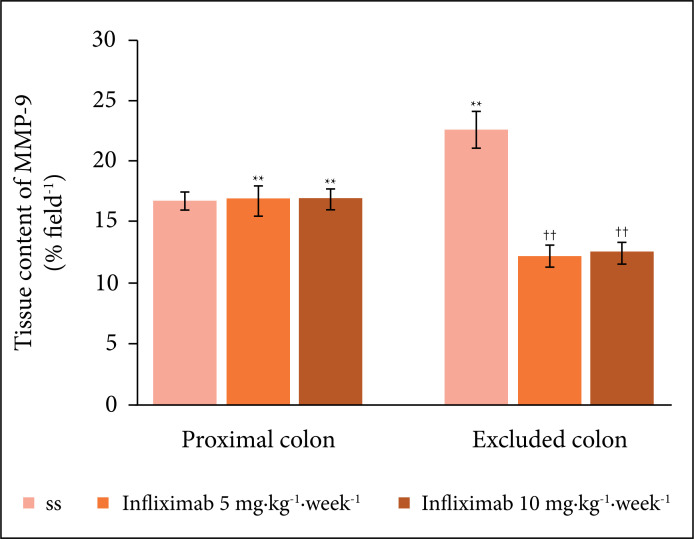
Tissue content of the MMP-9 protein in colic segments with and without intestinal transit in animals given an intervention with SS 0.9% or infliximab at dosages of 5 and 10 mg·kg^–1^·week^–1^ for five weeks. Note. ** = p < 0.01(SS 0.9% excluded colon > SS 0.9% proximal colon) (infliximab 5 and 10 mg·kg^–1^·week^–1^ proximal colon > excluded colon).†† = p < 0.01 (SS excluded colon 0.9% > excluded colon infliximab 5 and 10 mg·kg^–1^·week^–1^). Mann–Whitney test.

Figure 7 shows the values found for the tissue content of the MMP-9 protein in animals give a weekly intervention with SS 0.9% and infliximab at dosages of 5 and 10 mg·kg^–1^·week^–1^ for five weeks. It appears that there was a significant reduction in the tissue content of the enzyme in animals given an intervention with infliximab when compared to those that received SS 0.9%. There were no significant differences in the content of MMP-9 related to the dose of infliximab used.

## Discussion

Currently, HS is one of the most commonly used procedures in the daily practice of colorectal surgeons^7^^,^ 19 ^,^ 20. However, Hartmann himself drew attention to the risks related to attempts to reconstitute intestinal transit after the procedure^19^. It is estimated that half of the patients undergoing HS do not have their intestinal transit successfully restored and that the morbidity and mortality rates when reconstruction is performed are 50 and 10%, respectively^21^^-^ 24. The impossibility of reestablishing intestinal transit condemns these patients to live with the stoma and with a segment of rectum that is not functional for the rest of their lives^19^^,^ 24 ^,^ 25.

Different factors contribute to the high rates of morbidity and mortality after the reconstitution of intestinal transit in patients undergoing HS. It has been shown that dehiscence can occur in up to 5.7% of patients and is associated with morbidity and mortality rates of 42.8 and 4.9%, respectively^8^^,^ 24 ^,^ 25. These complications may be related to the clinical condition of the patients (great surgical risk, anemia, hypoalbuminemia) or the technical difficulty of performing an anastomosis in a segment of a defunctionalized rectum that has varying degrees of DC^25^. Surgical dehiscence after reconstitution of fecal transit is one of the complications most feared by the surgeon and, depending on the severity, requires a new derivation. Anastomoses performed on an inflamed intestinal wall, with increased activity of proteolytic enzymes, such as collagenases, have higher risks of dehiscence^26^. In rectal segments without fecal transit, there is an important reduction in the population of colonocytes followed by atrophy of the different layers that form the rectal wall, in addition to the development of a local inflammatory process with variable intensity^8^^,^ 27 ^,^ 28.

An experimental study evaluating the tissue content of collagen in an experimental model of DC, similar to that used in the present study, and showed that, in addition to the development of a mucosal inflammatory process, there is a significant reduction in the total collagen content in colic segments without fecal transit when compared to those with preserved traffic^11^. Using the Sirius red histochemical technique, which allows polarized light to distinguish between mature and immature collagen, these same authors showed that in the colon with fecal transit, there is a predominance of mature collagen, while in the colon without transit, there was immature collagen^11^. It is likely that these phenomena may be related to the lack of SCFA supply to the excluded intestinal transit segment, which reduces the synthesis and, consequently, the tissue collagen content in the excluded colon^29^^-^ 32.

Perhaps the reduction in collagen content in segments without fecal transit may also be related to the greater enzymatic destruction caused by the inflammatory process. With the greater infiltration of inflammatory cells, there is an increased production of collagenases and FOR, favoring the degradation of tissue protein^13^^,^ 26. This possibility is reinforced by the findings of an experimental study showing that the administration of SCFAs, particularly butyric acid and glutamine, in animals subjected to intestinal exclusion increased the tissue production of collagen, with a content similar to that found in the colon with preserved transit^9^^,^ 16 ^,^ 30 ^,^ 31.

The lack of a supply of SCFAs to the excluded colon modifies the cellular respiration mechanisms, increasing the production of FOR. Free oxygen radical oxidizes and denatures several proteins that make up the defense mechanisms of the colic epithelium. The destruction of these defense mechanisms allows the infiltration of antigens and bacteria into the intestinal lumen, triggering the inflammatory process in the excluded rectum. Short-chain fatty acids deficiency reduces the synthesis of proteins that make up the ECM, while worsening inflammation increases the production of proteolytic enzymes that destroy the ECM, including MMP-9.

Clear evidence of the importance of preserving the supply of SCFAs to the mucosa devoid of intestinal transit is the total recovery of the inflammatory process when fecal transit is restored^31^^-^ 33. It is possible that the decrease in collagen tissue content in the excluded colon is due to the lack of substrate for its synthesis, combined with the increased production of proteolytic enzymes by neutrophils, among them MMP-9, related to collagen degradation. This process may be one of the possible explanations for the increased risk of dehiscence of anastomoses performed on the excluded colon. Thus, reducing the inflammatory process in the segments could perhaps decrease these rates. However, no study has evaluated the total content of collagen or MMP-9 in exclusive segments of fecal transit, comparing colic segments with and without intestinal transit.

Infliximab has recently been used experimentally for the treatment of DC^15^. The antibody decreased the infiltration of inflammatory cells in the colic mucosa devoid of intestinal transit when it develops DC, significantly improving the tissue inflammatory process^15^. These findings are interesting because the reduction of neutrophilic infiltrate in colic segments without fecal transit could reduce the production of proteolytic enzymes, such as MMP-9, thereby increasing the content of tissue collagen. In other words, infliximab, which reduces the inflammatory process, at least from a theoretical point of view, could favor the healing of anastomoses performed on the excluded colon. The results found in the present study seem to confirm this possibility. It was found that in animals treated with infliximab, the inflammatory score in the colon devoid of fecal transit decreased significantly when compared to those treated with SS 0.9%. However, the importance of an adequate supply of SCFAs was confirmed when it was found that the inflammatory score was always significantly higher in the colon with no transit than in the colon with preserved fecal transit, regardless of the intervention substance used. When considering the segments without fecal transit, the results showed that there was a reduction in the inflammatory score in the animals that received infliximab, regardless of the dose used. This finding reinforces the importance of this drug in the treatment of inflammation of the excluded colon.

Regarding the total content of tissue collagen, the present study also showed the importance of maintaining an adequate energetic substrate for collagen synthesis. Regardless of the intervention substance used, the total collagen content did not change in colic segments with preserved traffic where the SCFAs supply was preserved. It should be noted that the collagen content was always higher in these segments than in colic segments without fecal transit, regardless of the intervention or the dose of infliximab used. In contrast, in colic segments without a SCFA supply, the application of infliximab at a dose of 10 mg·kg^–1^·week^–1^ increased the tissue collagen content compared to animals that received SS 0.9% or infliximab at a dose of 5 mg·kg^–1^·week^–1^; however, the collagen content was preserved in the colon. In other words, despite the reduction of the inflammatory process in the colon without intestinal transit in animals treated with infliximab and the increase in collagen, the lack of an energy supply for collagen synthesis provided by SCFA is an aspect that deserves consideration. It is possible that the provision of SCFA-rich solutions in the excluded colon, as shown in other studies, combined with the use of infliximab, may increase the tissue collagen content, making it closer to that of the colon with preserved transit^9^^,^ 16 ^,^ 29 ^,^ 30.

The results of this study also showed that in the colon with intestinal transit, the lowest inflammatory score was related to a stable content of MMP-9, regardless of the intervention substance used. Once again, the importance of a regular supply of SCFAs in preserving epithelial integrity, reducing neutrophilic infiltration and, consequently, reducing the production of proteolytic enzymes is a possibility to be considered. The MMP-9 enzyme is a protein that is, therefore, dependent on the cellular energy supply for its synthesis. In this study, its tissue content was not affected by the intervention used in the colon, as there was an adequate supply of SCFAs for its synthesis. Conversely, in the segments without fecal transit, with an increase in the inflammatory process and neutrophilic infiltration, there was a greater increase in the tissue content of MMP-9 in animals that received 0.9% SS. In those treated with infliximab, regardless of the dose used, there was a reduction in the tissue content of MMP-9. However, these values were lower than those found in the colon with preserved transit, reinforcing, once again, the importance of an adequate supply of SCFAs for the synthesis of proteins such as the MMP-9 enzyme itself.

The results of this study suggest that the administration of infliximab may be a useful strategy to reduce the mucosal inflammatory process and favor the healing process in exclusive segments of fecal transit that develop DC. It is possible that these effects may be even more relevant when combining applications of SCFA-containing enemas with infliximab.

Finally, it is important to highlight the limitations of the present study. This is an experimental study that was carried out with a small number of animals. Therefore, it is prudent to remember that clinical studies are still needed to extrapolate the findings of this experimental study to humans.

## Conclusion

In the DC model proposed in the present study, intervention with infliximab reduced the inflammatory process of the mucosa excluded from intestinal transit, increased the total collagen content and decreased the tissue content of MMP-9. The content of tissue collagen in the DC colon without intestinal transit was found to be increased after the application of infliximab at higher concentrations.
